# Report of Army Committee. U. S. V. M. A.

**Published:** 1895-11

**Authors:** J. P. Turner

**Affiliations:** Chairman; Ft. Myer, Va.


### REPORT OF THE COMMITTEE ON ARMY LEGISLATION OF THE UNITED STATES VETERINARY MEDICAL ASSOCIATION.

I HAVE the honor to submit herewith the report of the Committee on Army Legislation, and as usual we can report no progress. Ever hopeful of success, we endeavored to get Congress to take some action in our behalf during the last session, but after making a thorough investigation, and taking the advice of several politicians in Washington, we decided to relinquish our struggle and await the action of a more favorable Congress. Your Chairman was in Washington during the entire term of the last Congress, but could not get any favorable action on the army bill, owing to the struggle with the silver problem. There was great difficulty in getting the regular appropriation bills passed, and all bills calling for extra appropriations were promptly killed, unless they were political in their nature, hence we decided that it would be preferable to allow our bill to remain in the committee-room unacted upon than have it killed upon the floor of the House.

Your Chairman, in conjunction with a Committee of Army Veterinary Surgeons, has made the preliminary arrangements for the introduction of a new bill next winter (1895), which will be the first regularly organized efforts made by both bodies. We have reasons to believe that the War Department will assist our bill, as a new Commanding General was appointed September 30th, and we believe he will be as friendly to us as the retiring General.

While making inquiries during the last session of Congress, your Committee learned that it would have been possible to have obtained army legislation several years ago had the proper methods been resorted to, and harmony existed between the Army Veterinary Surgeons and certain active civilian practitioners. This want of harmony has caused defeat more than once, and acting on the knowledge that it is far easier to kill legislation than to cause it, this mistake should never be made again.

A bill was introduced organizing a corps of commissioned veterinary officers ranking from lieutenant to major. This was a move in the proper direction, but the reorganization scheme was almost too ambitious, and met with a great deal of opposition from the line officers of the army, and it was especially unpopular to three or four of the army veterinary surgeons of the "old school," although it met with the approval of many of the corps. This bill was to reorganize, or rather to organize a veterinary corps, and incidentally provide comfortable berths for those who pushed it along. For the veterinary profession at large it was very pleasing to see the profession thus raised and honored by the Government, but to those men who had never received a veterinary college education, owing to the fact that there were no colleges in this country at the time of their entrance into the profession, and who had served their country as army veterinary surgeons in those trying days of the fifties and sixties, it was anything but right and justice to grant them three months' leave of absence in which to brush up their studies, and be pitted against newly graduated veterinarians, and not have their long and arduous services count for anything in their favor. Knowing that the gentleman who was ambitious to be the chief veterinarian under the new measure had put himself on record as being unfavorably disposed toward them, is it any wonder that these old men, who have had from twenty-five to thirty-five years of service, should protest against its passage and virtually kill the bill?

Two members of your committee have seen army service and understand the conditions thoroughly. It is their opinion, backed up by the opinions of hundreds of army officers, that all reforms in the veterinary corps of the army must be very gradual and not excite opposition from the army, and must be not only for the good of the service, but it must protect in a measure those men of long service in the army. A mistake made in many previous attempts at legislation has been the introduction of bills with many conditional clauses and retirement and pension features. A simply-framed bill has far more chances of success than a long-drawn-out bill, and a bill which partakes of a pension nature will surely be killed by the present Congress, hence your committee, acting in accord with a commitee of army veterinary surgeons, has decided to introduce the following bill this winter, and in its behalf we earnestly ask your assistance:

"Be it enacted by the Senate and House of Representatives of the United States in Congress assembled:

"That the U. S. army veterinary surgeons be given the rank, pay, and allowances of second lieutenants mounted."

If such a bill be passed it will retire two or three of the old men in the service, and will protect them, inasmuch as the term "allowance" will be interpreted to include retirement and pension, and these lesser terms not being included in the language of the bill are not so apt to excite the opposition of Congress. The passage of this bill would be an entering wedge for vast improvements, and once recognized our future success is assured.

Finally, we must add that the greatest stumbling-block to success with our bills in the past has been the lack of funds to back them up, for without funds your committee is powerless in these days of practical politics. In closing our report we most respectfully request that some appropriation be passed by your body, in order that your committee may have an opportunity to aid the army veterinary surgeon when help is most needed. I am, sir,

Very respectfully, J. P. TURNER,
FT. MYER, VA., September 1, 1895. Chairman.

